# β-hCG resolution times during expectant management of tubal ectopic pregnancies

**DOI:** 10.1186/s12905-015-0200-7

**Published:** 2015-05-21

**Authors:** D. Mavrelos, M. Memtsa, S. Helmy, G. Derdelis, E. Jauniaux, D. Jurkovic

**Affiliations:** Institute of Women’s Health, University College London, London, UK; Gynaecological Diagnostic and Outpatient Treatment Unit, University College London Hospital, Lower Ground Floor, Elizabeth Garrett Anderson Wing, 250 Euston Road, London, NW1 6BU UK; University Hospital, Vienna, Austria

**Keywords:** Pregnancy, Ectopic, Expectant management

## Abstract

**Background:**

A subset of women with a tubal ectopic pregnancy can be safely managed expectantly. Expectant management involves a degree of disruption with hospital visits to determine serum β-hCG (β-human chorionic gonadotrophin) concentration until the pregnancy test becomes negative and expectant management is considered complete. The length of time required for the pregnancy test to become negative and the parameters that influence this interval have not been described. Information on the likely length of follow up would be useful for women considering expectant management of their tubal ectopic pregnancy.

**Methods:**

This was a retrospective study at a tertiary referral center in an inner city London Hospital. We included women who were diagnosed with a tubal ectopic pregnancy by transvaginal ultrasound between March 2009 and March 2014. During the study period 474 women were diagnosed with a tubal ectopic pregnancy and 256 (54 %) of them fulfilled our management criteria for expectant management. A total of 158 (33 %) women had successful expectant management and in those cases we recorded the diameter of the ectopic pregnancy (mm), the maximum serum β-hCG (IU/L) and levels during follow up until resolution as well as the interval to resolution (days).

**Results:**

The median interval from maximum serum β-hCG concentration to resolution was 18.0 days (IQR 11.0–28.0). The maximum serum β-hCG concentration and the rate of decline of β-hCG were independently associated with the length of follow up. Women’s age and size of ectopic pregnancy did not have significant effects on the length of follow up.

**Conclusion:**

Women undergoing expectant management of ectopic pregnancy can be informed that the likely length of follow up is under 3 weeks and that it positively correlates with initial β-hCG level at the time of diagnosis.

## Background

Tubal ectopic pregnancy has traditionally been perceived as a potentially life threatening condition with significant risk of serious maternal morbidity and mortality. In recent years, advances in ultrasound imaging and the availability of sensitive urinary pregnancy test have facilitated the diagnosis and management of small ectopic pregnancies with limited potential to grow, cause harm. The majority of these ectopic pregnancies tend to resolve spontaneously without any complications and need for intervention [1]. As a result, expectant management has been recognized as an acceptable option for many women with a tubal ectopic. We have previously shown that, given certain criteria, up to one third of women diagnosed with a tubal ectopic pregnancy by high resolution transvaginal ultrasound can be managed expectantly without any intervention, thus sparing these patients the morbidity of surgery and side effects of medical treatment [2].

However, experience with expectant management is limited and little is known of the natural history of pregnancies managed in this way. Previous studies have focused on assessment of β-hCG clearance curves in women following treatment with methotrexate or after salpingotomy [3]. In these clinical situations slower than expected β-hCG clearance often triggers additional medical or surgical treatment in order to minimize the risk of complications.

Appropriate selection of women is the key factor to determine the safety and success rate of expectant management. Counseling of women who contemplate expectant management also needs to include evidence-based information about the likely length of time required for β-hCG levels to decline to pre-pregnancy levels. At present there is no information available regarding the clearance curves in women with tubal ectopics managed expectantly. The aim of this study was to investigate a large group of women on our expectant management protocol in whom ectopic pregnancies resolved spontaneously and define the length of time required for β-hCG to drop below the detection level of the serum bioassay or the urine test.

## Methods

This was a retrospective study of women diagnosed with a tubal ectopic pregnancy and managed expectantly at the Early Pregnancy Unit (EPU) at University College London Hospital between March 2009 and March 2014.

Women attending the EPU with early pregnancy complications undergo clinical examination and a transvaginal scan using a high frequency transvaginal probe (Voluson GE E8) in order to establish the location and viability of the pregnancy. The examination is performed systematically in the following manner: first the uterus is examined in the transverse plane to identify the cervical canal, the uterine cavity and both interstitial portions of the fallopian tubes. Acquired uterine anomalies, such as fibroids or adenomyosis, are diagnosed based on direct visualisation using previously described diagnostic criteria [4, 5]. The ovaries are routinely visualised and examined for the presence of corpora lutea or any detectable ovarian pathology. The pouch of Douglas is examined for the presence of blood. A diagnosis of tubal ectopic pregnancy is made when a swelling with typical ultrasound appearances is seen in either adnexa, separate from the ovary and the corpus luteum. According to their morphological characteristics ectopics are classified into five different categories: (1) inhomogeneous solid swelling, (2) empty gestation sac, (3) gestation containing a yolk sac, (4) gestational sac containing an embryo with no cardiac activity, (5) gestational sac containing a live embryo.

Women with a diagnosis of an ectopic pregnancy complaining of moderate or severe pelvic pain, those showing clinical signs of cardiovascular instability and those with evidence of significant haemoperitoneum on ultrasound scan, which is defined by the presence of blood clots in the pouch of Douglas, were immediately admitted for emergency surgery. Asymptomatic or minimally symptomatic women with evidence of a live ectopic pregnancy on ultrasound scan are also routinely offered emergency surgical treatment. Minimal symptoms are defined as those that do not in any way interfere with activities of daily living.

All other women with ultrasound diagnosis of ectopic pregnancies have a blood sample taken for serum β-hCG and progesterone measurements. The results are typically available on the same day, 2 to 6 h later. Women are provided with a leaflet describing the significance of ectopic pregnancy to their health and available management options. In all women the decision whether to manage ectopic pregnancy expectantly is made on the same or the following day. Women with initial serum β-hCG <1500 IU/L are offered expectant management. Women with hormone readings above these levels and those who opted against expectant management are also offered surgery. Surgery is also offered to women who are unable to comply with follow up or whose command of English is severely limited. We do not use methotrexate for the management of tubal ectopic pregnancy in our unit except in women who consented to participate in ongoing randomized trials on the management of ectopic pregnancies. These women were excluded from data analysis.

All women selected for expectant management are managed on outpatient basis. They are advised not to travel, to avoid sexual intercourse and to return to the clinic if they experience a significant increase in abdominal pain. Women are then followed up with serial blood tests until the serum β-hCG declines to <20 IU/L or their urine pregnancy test becomes negative. The protocol is individualized in each case so that the interval of repeat serum β-hCG test varies between 2 and 7 days. Expectant management is discontinued if women opt out form further follow up or if they experience significant increase in abdominal pain. Women in whom serum β-hCG levels increases to ≥ 2000 IU/L during follow up or show sustained rise on repeated measurements are also advised to discontinue expectant management and opt for surgery.

We searched our clinical database for women who had been diagnosed with a tubal ectopic pregnancy, opted for expectant management and completed their follow-up. We recorded the median diameter of the tubal ectopic pregnancy at the initial visit. We also recorded all β-hCG measurements between the initial visit and until follow-up was completed. We identified the maximum serum β-hCG concentration during follow up (β-hCG_max_ [IU/L]) and β-hCG concentration at the first determination after the maximum(β-hCG_sub_ [IU/L]). We calculated the daily β-hCG change: β-hCG_trend_ [%change/day]) between β-hCG_max_ and β-hCG_sub_ as follows:

$$ \upbeta \hbox{-} \mathrm{h}\mathrm{C}{\mathrm{G}}_{\mathrm{t}\mathrm{rend}}=\left(\left(\upbeta \hbox{-} \mathrm{h}\mathrm{C}{\mathrm{G}}_{\mathrm{sub}}\hbox{--} \upbeta \hbox{-} \mathrm{h}\mathrm{C}{\mathrm{G}}_{\max}\right)/\upbeta \hbox{-} \mathrm{h}\mathrm{C}{\mathrm{G}}_{\max}\right)/\left({\mathrm{t}}_{\mathrm{sub}}\hbox{-}\ {\mathrm{t}}_{\max}\right). $$

The time until β-hCG complete clearance (t_res_ [days]) was defined as the time from β-hCG_max_ to a serum β-hCG < 5 IU/L.

We used the Shapiro-Wilk test to assess normality of variables’ distribution. None were normally distributed and they were transformed using Box-Cox transformation. We used Pearson statistic to assess bivariate correlation. We constructed a linear regression model to assess predictive variables’ independence. We used transformed t_res_ (days) as independent variable while the transformed serum β-hCG_max_ (IU/L), median diameter of tubal ectopic pregnancy (mm) and β-hCG_trend_ (IU/L) were dependent variables. The level of significance was <0.05 throughout. The model selection method was forward stepwise with *p* < 0.05 threshold for inclusion and *p* > 0.10 for exclusion. SPSS 22 (IBM Corp) was used for statistical analysis.

### Details of ethics approval

We were advised by the Joint Research Office of University College London and University College London Hospital that as long as patient identifiable data were not seen by anyone outside the clinical care team formal ethics approval was not needed for this retrospective study.

## Results

During the study period 19,056 women with suspected early pregnancy complications attended our unit and 474 women (2.5 %, 95 % CI 2.3–2.7) were diagnosed with a tubal ectopic pregnancy. In this group, 218/474 (46.0 %, 95 % CI 41.5–50.5) had immediate surgical management whilst 256/474 (54 %, 95 % CI 49.5–58.5) were considered suitable for expectant management. A sub-group of 30/256 women (11.7 %, 95 % CI 7.8–15.6) were recruited for a randomized controlled trial (RCT) comparing methotrexate therapy versus conservative expectant management and these women were excluded from the data analysis.

**Fig. 1:**
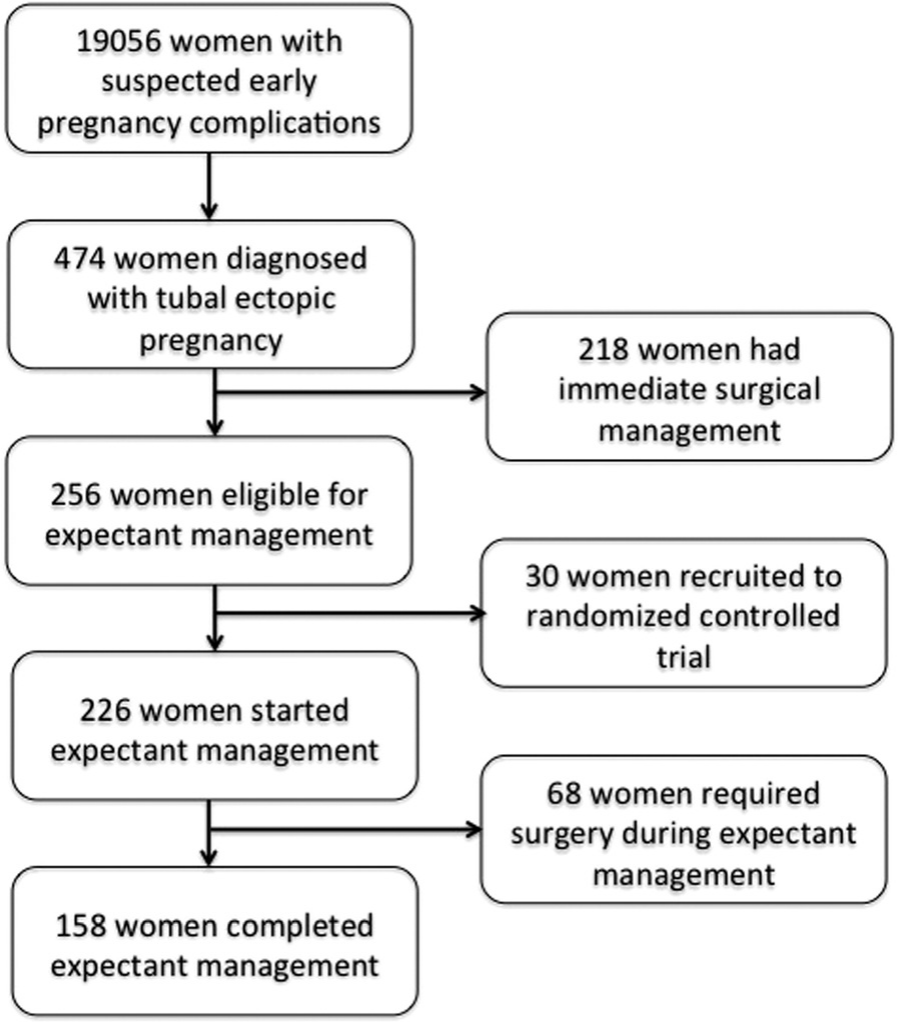
Chart depicting the flow of patients through the study. During the study period 19,056 women were seen, 474 were diagnosed with a tubal ectopic pregnancy, 226 women entered expectant management and 158 completed expectant management of tubal ectopic pregnancy without any intervention

68/226 women (30 %, 95 % CI 24.0–36.0) were unsuccessful in expectant management of their tubal ectopic pregnancy and required surgery according to our protocol. The final study group included 158 out 226 women (70.0 %, 95 % CI 64.0–76.0) with a tubal ectopic pregnancies who were successfully managed expectantly (Fig. 1). This accounted for 33 % (95 % CI 29.0–37.5) of all ectopic pregnancies diagnosed during the study period. The median age of women in this study was 33 years (IQR 29–37). The median diameter of tubal ectopic pregnancy was 14.0 mm (IQR 6.0–30.45), the median number of β-hCG determinations was 5 (IQR 4–7) and the median initial serum β-hCG concentration was 306 IU/L (IQR 136.75–658). In 109 cases (69.0 %, 95 % CI 61.8–76.2), the initial β-hCG represented the maximum recorded reading whilst in the remaining 49 cases (31 %, 95 % CI 23. 8–38.2) the readings continued to increase during the first follow - up visit. The median peak serum β-hCG_max_ concentration was 393.5 IU/L (IQR 152.75–756.5) and the median β-hCG_trend_ was −11.3 %/day (IQR −18.8– −5.3).

The median length of follow-up from presentation was 20 days (IQR 11–31). The 90^th^ and 95^th^ percentiles for length of follow-up from presentation were 45 and 56 days respectively. Median t_res_ was 18 days (IQR 11–28). The 90^th^ and 95^th^ percentiles for t_res_ were 37 and 50 days respectively.

**Fig. 2:**
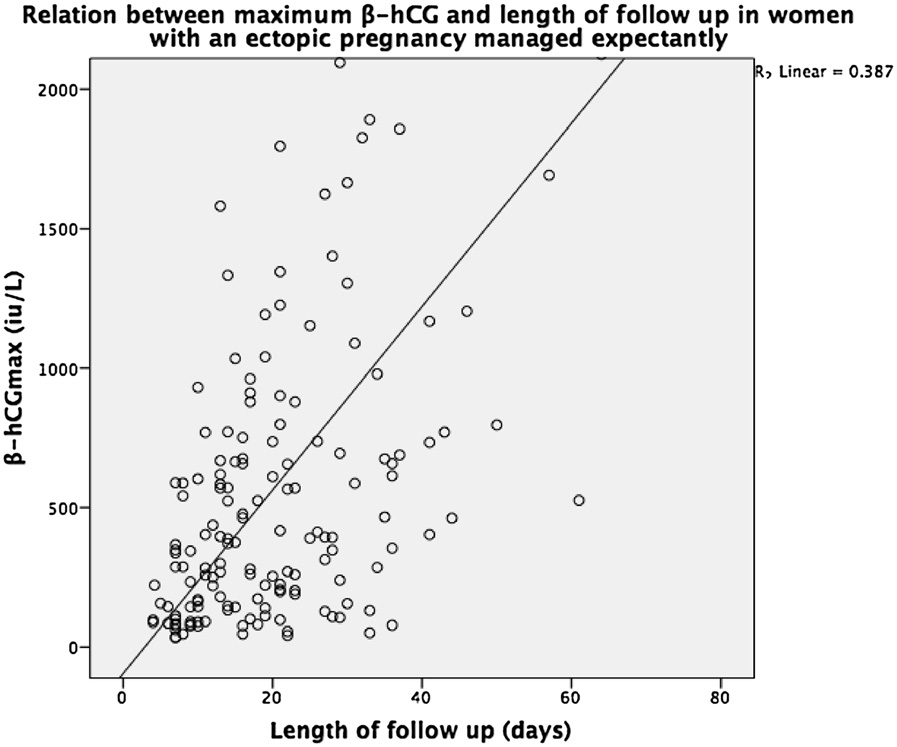
Relation between maximum β-hCG during follow up and length of follow up in women diagnosed with a tubal ectopic pregnancy managed expectantly

**Fig. 3:**
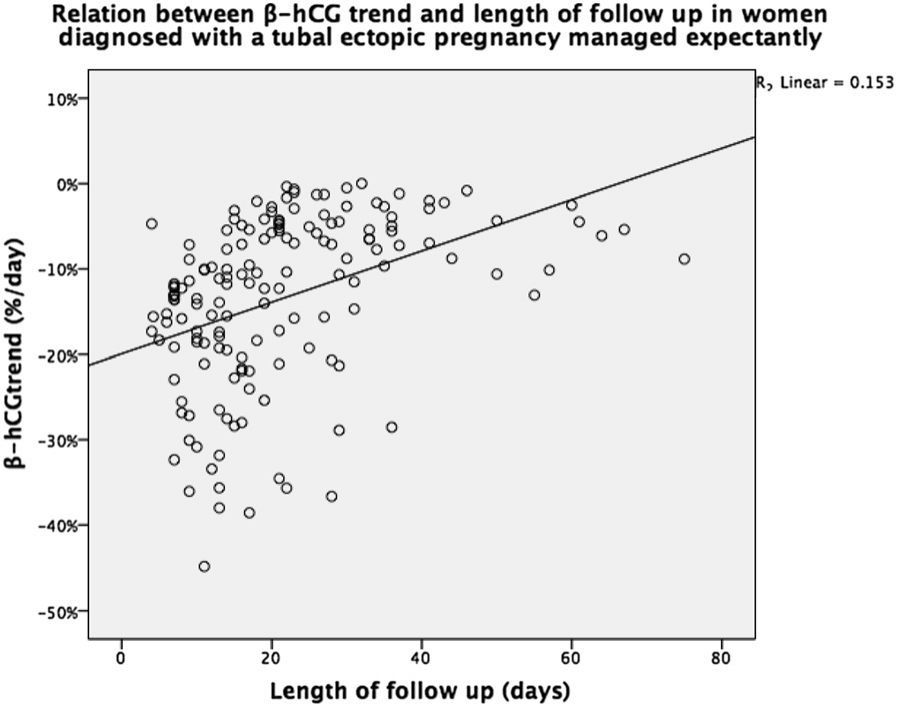
Relation between β-hCG trend and length of follow up in women diagnosed with a tubal ectopic pregnancy managed expectantly

**Fig. 4:**
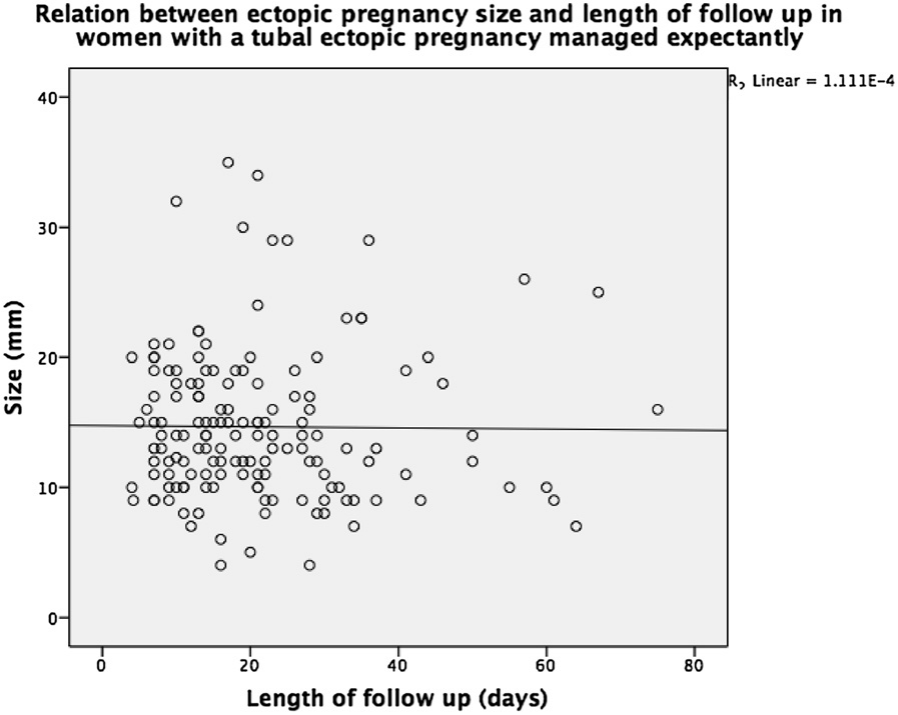
Relation between ectopic pregnancy size and length of follow up in women diagnosed with a tubal ectopic pregnancy managed expectantly

The relation between β-hCG_max_, β-hCG_trend_, ectopic pregnancy size and length of follow-up is shown in Figs. 2, 3 and 4. There was a significant positive correlation between t_res_ and β-hCG_max_ and β-hCG_trend_. There was no significant correlation between t_res_ and woman’s age or ectopic pregnancy diameter at presentation (Table 1).

**Table 1:** Univariate and multivariate regression coefficients between the length of time required for β-hCG to return to pre pregnancy levels and age, β-hCG_max ,_β-hCG_trend_ and ectopic pregnancy size in women with a tubal ectopic pregnancy undergoing expectant management

	Unadjusted coefficient (95 % CI)	Adjusted coefficient (95 % CI)	Sig.
Age	0.042 (−0.041–0.071)	−	ns
β-hCG_max_	0.520 (0.384–0.655)	0.533 (0.418–0.647)	0.01
β-hCG_trend_	0.444 (0.303–0.586)	0.459 (0.345–0.574)	0.01
Size	−0.12 (–0.060–0.052)	−	ns

The linear regression model retained both transformed β-hCG_max_ and transformed β-hCG_trend_ as independent predictors of transformed t_res_. The adjusted coefficients are presented in Table 1. The adjusted R^2^ for the linear regression model was 0.454.

## Discussion

Our data showed that in women with tubal ectopic pregnancies selected for expectant management the median interval for β-hCG to return to pre-pregnancy levels is 20 days from the initial presentation or 18 days after reaching the peak. During this period 90 % women are likely to need to attend for 5 additional hospital visits including blood tests.

In comparison to previous studies on expectant management of tubal ectopic pregnancies the time required for β-hCG resolution in our study was shorter. In a placebo controlled randomized trial on the use of methotrexate in ectopic pregnancies, Korhonen et al. [6] found median resolution time of 24 days in their placebo group. Ylostalo et al. [7] reported average resolution times of 22 days in women managed expectantly. Both these studies were more liberal than ours in their selection criteria and included women with β-hCG levels up to 5000 IU/L. These longer reported β-hCG resolution times support our finding that the length of follow up is increased in women who present with higher peak β-hCG concentrations. In the recently published randomized controlled trial of expectant management vs. methotrexate [8] the length of follow up was considerably longer for both arms, (38 vs. 34) days despite only marginally more liberal inclusion criteria than those in our study (max β-hCG less than 2000 IU/L). Majority of women in this study were diagnosed with pregnancies of unknown location (PUL) with plateauing serum β-hCG levels. It is likely that most of these PULs were slowly resolving incomplete miscarriages which may explain relatively long resolution times. In addition, women in that study had weekly β-hCG determinations, which may have contributed, to the length of follow up. It is possible that our study suffered from a similar bias, overestimating the true β-hCG clearance time as once a declining β-hCG is established, the frequency of determinations decreased meaning that the true time of clearance is missed. Nevertheless our results remain clinically useful guide for both patients and clinicians.

Clearance time showed considerable variation in our study ranging from few days up to several weeks. However, more than 90 % of ectopics had resolved by the end of the 7^th^ week of follow up. As expected, the maximum β-hCG attained during follow up and the rate of decline from the peak significantly influenced the length of necessary follow up. These two variables are measures of the amount of trophoblastic tissue present in the Fallopian tube and its vitality and so can be used to inform patients about the likely follow up ahead. In contrast, we found that ectopic pregnancy diameter does not give any information about the length of follow-up required. This is possibly because, according to our own observations, the measured diameter reflect varying degrees of oedema and hematosalpinx and so do not correlate closely with the amount of active trophoblastic tissue.

A previous RCT of systemic methotrexate vs. salpingostomy, employing similar selection criteria as our study, found a median follow up for the medical treatment arm of 19 days [9]. Another more recent study assessed the value of the epidermal growth factor receptor inhibitor gefitinib as an adjunct to methotrexate for the treatment of women with tubal ectopic pregnancy [10]. In that study, the overall median resolution time for women receiving combination therapy was 21 days. In the subgroup of women with a maximum β-hCG ≤ 1500 IU/L the median resolution time was 18 days which is identical to our study (*n* = 6). A larger prospective study is therefore needed to determine whether gefitinib will offer any benefit in terms of shortening resolution time in women receiving medical treatment for tubal ectopic pregnancy. While we have included all cases in our database that met the inclusion criteria, thereby minimizing the risk of selection bias, our study was retrospective which will limit the generalizability of our results. We aim to validate our findings in a prospective study.

## Conclusion

Women undergoing expectant management of ectopic pregnancy can be informed that the likely length of follow up is under 3 weeks and that this length of this interval positively correlates with the initial level of β-hCG at the time of diagnosis. The findings of our study will be useful to women and clinicians when considering expectant management of tubal ectopic pregnancies. Information about the success rate, the likely length of follow up and number of visits to hospital should help women to make informed choices about their care. This may help to facilitate wider use of expectant management of ectopic pregnancy which use is currently limited to a handful of centres worldwide.
